# Application of moisturizer to neonates prevents development of atopic dermatitis

**DOI:** 10.1186/1939-4551-8-S1-A252

**Published:** 2015-04-08

**Authors:** Kumiko Morita

**Affiliations:** 1 National Center for Child Health and Development, Japan

## Background

Skin barrier dysfunction contributes to development of atopic dermatitis (AD). We performed a prospective, randomized controlled trial to investigate whether protecting the skin barrier with a moisturizer prevents development of AD and allergic sensitization.

## Methods

We enrolled 118 neonates at high risk for AD (based on family history of AD in parents or siblings) and randomized to intervention group or control group (59 infants for each group). In intervention group, we applied emulsion-type moisturizer daily on a whole body during the first 32 weeks of life. All infants were scheduled to visit at weeks 4, 12, 24 and 32 of life and were examined the skin condition. The onset of AD (eczematous skin lasting more than 4 weeks) and eczema (lasting more than 2 weeks) were assessed by a blinded dermatologist, based on the modified Hanifin and Rajka criteria. The primary outcome was the cumulative incidence of AD plus eczema (AD/eczema) at 32 weeks. The secondary outcome, allergic sensitization, was evaluated based on serum levels of allergen-specific IgE, measured by high-sensitivity allergen microarray of diamond-like carbon-coated chip (UMIN Clinical Trials Registry Identifier: UMIN000004544).

## Results

Among 118 infants, 47 developed AD/eczema (19 in the intervention and 28 in the control group). The cumulative incidence of AD/eczema was approximately 32% fewer in infants randomized to intervention group than that in controls by week 32 (P=0.012 in log-rank test). We failed to reveal a statistically significant effect of emollient-use on allergic sensitization based on the level of IgE antibody against egg white at 0.34 kUA/L CAP-FEIA equivalents. However, the sensitization was significantly higher in infants who developed AD/eczema than in those who did not (odds ratio, 2.86; 95% confidence interval, 1.22-6.73).

## Conclusions

Daily application of moisturizer during the first 32 weeks of life reduces the risk of AD/eczema in high-risk infants. Allergic sensitization during this time period is associated with the presence of eczematous skin, but not with moisturizer use (J Allergy Clin Immunol. 2014; in press).

